# N6-Methylandenosine-Related lncRNA Signature Is a Novel Biomarkers of Prognosis and Immune Response in Colon Adenocarcinoma Patients

**DOI:** 10.3389/fcell.2021.703629

**Published:** 2021-07-15

**Authors:** Peiling Zhang, Guolong Liu, Lin Lu

**Affiliations:** Department of Medical Oncology, Guangzhou First People’s Hospital, School of Medicine, South China University of Technology, Guangzhou, China

**Keywords:** colon adenocarcinoma, M6A, lncRNA, tumor immune microenvironment, prognosis

## Abstract

**Background:**

Colon adenocarcinoma (COAD) is the most common type of colon cancer. To date, however, the prognostic values of m6A RNA methylation-related long non-coding RNAs (lncRNAs) in COAD are largely unknown.

**Materials and Methods:**

The m6A-related lncRNAs were identified from The Cancer Genome Atlas (TCGA) data set. Univariate and multivariate Cox regression analyses were performed to explore the prognostic m6A-related lncRNAs. Consistent clustering analysis was performed to classify the COAD patients into different subgroups based on the expression of m6A-related lncRNAs. The potential biological functions as well as differences in the stemness index and tumor immune microenvironment between different subgroups were analyzed. The prognostic m6A-related lncRNAs were used to establish an m6A-related lncRNA risk model to predict prognosis and survival status.

**Results:**

We identified 31 m6A-associated lncRNAs with prognostic values from the TCGA data set. Based on the expression of prognostic m6A-associated lncRNAs, TCGA-COAD patients were classified into three clusters using consistent clustering analysis. There was a low correlation of tumor stemness between the three clusters but a significant correlation with the tumor immune microenvironment as well as the tumor mutational load. Thirty-one prognostic-related m6A-associated lncRNAs were used to construct a risk model, which was further determined by survival analysis, receiver operating characteristic (ROC) curve, and univariate and multifactor Cox analysis. The m6A-related risk model demonstrates good performance in predicting prognosis and survival status. The model-based high-risk group exhibited poorer overall survival (OS) compared with the low-risk group.

**Conclusion:**

In this study, we construct a risk model that consists of 31 m6A-related lncRNAs with independent prognostic values in COAD. Our study shows the critical roles of these 31 m6A-related lncRNAs in the tumor immune microenvironment, indicating the prospect of informing prognostic stratification and the development of immunotherapeutic strategies for COAD patients.

## Introduction

Colon cancer is the third most common type and the second cause of cancer-related death in the world (Siegel et al., 2020). Among all the pathological subtypes, more than 90% of colorectal cancers are adenocarcinoma derived from epithelial cells of the colorectal mucosa (COAD) (Hamilton et al., 2000; Rawla et al., 2019). The current major strategies for COAD include surgery, chemotherapy, and radiotherapy. Recently, targeted therapies and immunotherapies have achieved big advancements, but the survival rate of COAD patients remains unsatisfactory (Kekelidze et al., 2013). The unsatisfactory outcomes might be due to most of the patients being diagnosed at late stages and more prone to develop distant metastasis. The 5-year relative survival rate of colorectal cancer ranges from 90% of patients diagnosed with the localized disease to 14% of patients diagnosed with the distant-stage disease (Siegel et al., 2020). Therefore, it is of great importance to identify more specific biomarkers for the diagnosis and treatment in COAD.

Long non-coding RNAs (lncRNAs), as non-protein-coding transcripts longer than 200 nucleotides (nt), modulate various cellular processes, including tumor progression and immune cell infiltration (Yao et al., 2019; Di Martino et al., 2021). Dysregulation of lncRNAs plays key roles in various cancers, such as glioblastoma, gastric cancer, non-small cell lung cancer, colon cancer, etc. (Zhu et al., 2018; Lan et al., 2019; Sun et al., 2019; Chen et al., 2020; Hong et al., 2020; Yi et al., 2020). For example, lncRNA ROR1-AS1, as an oncogene, promotes colon cancer cell proliferation (Wang X.Y. et al., 2020). Silencing LINC00460 can reduce the expression of ANXA2 by upregulating miR-433-3p, thereby inhibiting cell invasion in colon cancer (Hong et al., 2020).

However, the mechanisms of regulating the expression of lncRNAs are largely unknown. Several studies indicate that lncRNAs could be regulated by N6-methyladenosine (m6A) modification (Fazi and Fatica, 2019; Dai et al., 2020; He et al., 2020; Yi et al., 2020). N6-methyladenosine (m6A), which is considered the most abundant apparent methylation modification in messenger ribonucleic acid (mRNAs) and non-coding ribonucleic acid, plays crucial roles in almost all stages of RNA metabolism, including RNA splicing, nuclear export, translation decay, expression, etc. The m6A modification is a reversible dynamic RNA epigenetic process, which is regulated by three types of m6A regulators, including methyltransferase “writers” (METTL3, METTL14, WTAP, METTL16, etc.), demethylase “erasers” (FTO and ALKBH5), and m6A binding protein “readers” (YTHDC, YTHDF1/2/3, etc.) (Yang et al., 2018; Lan et al., 2019; Zaccara et al., 2019). Dysregulated expression of m6A enzymes could regulate the cellular function and tumor microenvironment (TME) of tumors (Han et al., 2020; Zhang et al., 2020; Chong et al., 2021).

Both m6A enzymes and lncRNAs are ideal diagnostic and prognostic markers. Accumulating evidence shows that m6A-related mRNAs and lncRNAs can serve as novel potential targets to predict the prognosis for multiple cancers (Barros-Silva et al., 2020; Li et al., 2020; Meng et al., 2020; Xu et al., 2020, 2021; Jin et al., 2021). For instance, METTL14 is a prognosis-associated regulator of m6A RNA methylation in hepatocellular carcinoma (Li et al., 2020). Patients with pancreatic cancer accompanied by genetic alterations in m6A regulators have worse disease-free and overall survival (OS) (Meng et al., 2020). m6A writer VIRMA regulates the expression of oncogenic lncRNAs CCAT1 and CCAT2 in prostate cancer by affecting their stability and abundance (Barros-Silva et al., 2020). However, the expression and biological functions of m6A-related lncRNAs in COAD are largely unknown.

In this study, we constructed an m6A-related prognostic lncRNA model in COAD according to The Cancer Genome Atlas (TCGA) database. The correlations between the m6A-related lncRNA model and tumor immune microenvironment as well as immune cells are explored. Moreover, the performance of the m6A-related lncRNA model is verified.

## Materials and Methods

### Processing of Data Sets

For the TCGA-COAD cohort, all data, including mRNA sequencing data, lncRNA sequencing data, mutation data, and corresponding clinical information, were downloaded from the TCGA website^1^. It excludes patients without survival information from further evaluation. The relevant colon cancer data sets were downloaded from the GEO database^2^ for further analysis, including seven eligible data sets GSE110224, GSE14333, GSE29621, GSE37892, GSE41328, GSE64857, and GSE75316. All the GEO data were normalized using the sva package (Leek et al., 2012).

### Screening for Differentially Expressed m6A RNA Methylation Regulators

We extracted the expression matrix of 23 m6A RNA methylation regulators based on one previous article (Zaccara et al., 2019), including writers (METTL3, METTL14, METTL16, WTAP, VIRMA, RBM15, RBM15B, and ZC3H13), erasers (FTO and ALKBH5), and readers (YTHDC1, YTHDC2, IGF2BP1, IGF2BP2, IGF2BP3, YTHDF1, YTHDF2, YTHDF3, HNRNPC, LRPPRC, HNRNPA2B1, FMR1, and RBMX). The differentially expressed m6A regulators were analyzed on the sangerbox website^3^ using the R package “limma” for variance analysis. The results are demonstrated using plotting volcano and violin plots (Ritchie et al., 2015; Villanueva et al., 2016; Adler and Kelly, 2020). Meanwhile, correlation analysis was performed by the sangerbox website, using Pearson to calculate correlation coefficients.

### Screening for Differentially Expressed m6A-Associated lncRNA

LncRNA annotation files of the human genetic reference genome GRCh38 were downloaded from the GENCODE website^4^ to annotate the lncRNA sequencing data downloaded from TCGA. Differentially expressed lncRNAs were screened using the “limma” package and plotted using a volcano map on R software. Pearson correlation analysis was first implemented for mining m6A-related lncRNAs (with the | Pearson *R*| > 0.4 and *p* < 0.001). The direct correlation regulatory network of m6A genes with lncRNAs was visualized using Cytoscape software (Otasek et al., 2019).

### Consistent Clustering of m6A-Associated lncRNAs

Cluster consistency clustering analysis based on the expression of 31 prognostic m6A-associated lncRNAs classified patients into three groups based on the best *k*-means clustering by using the ConsensusClusterPlus R package (Wilkerson and Hayes, 2010). The Kaplan–Meier method was used to calculate the OS rate between different clusters. We also performed principal component analysis (PCA) to obtain the validity of the consensus clusters with intuitive signatures of the three clusters (Abdi and Williams, 2010). A heat map was plotted by online platform Bioinformatics^5^.

### Tumor Stemness

A prediction model was developed by Malta et al. (2018) by using the OCLR algorithm to calculate the dryness index of tumors, mainly including mRNAsi and mDNAsi. The degree of tumor stemness between different subgroups was compared by the stemness index of samples, which was calculated by downloading the R package from Github^6^. To further explore the differences in tumor stemness degree among multiple clusters, we analyzed the current stem cell markers used to identify and localize cancer stem cell (CSC) subpopulations in colorectal cancer. The Kruskal test was used to compare the differences between different subgroups.

### Comparison of the Immune Microenvironment in Different Clusters

To compare the TME between different clusters, first, the “estimate” package was used to calculate the estimated, immune, and matrix fractions for further analysis of TME (Becht et al., 2016). To explore whether there were any differences in immune genes between different clusters, the immunological signature gene set was downloaded from the Msigdb website^7^ to compare the differences in immune genes between various clusters. Algorithms were used to explore the extent of immune cell infiltration in the three subgroups. On the one hand, we used the CIBERSORT algorithms to assess the proportion of 22 immune cell subtypes based on TCGA-COAD samples at the online website^8^ (Newman et al., 2019). On the other hand, we directly uploaded the TCGA-COAD gene expression matrix on the xcell website^9^ to obtain the proportion of 64 immune cell types (Aran et al., 2017). The Kruskal test was used to compare the differences between these three subgroups.

### Calculation of Prognostic Risk Scores and Clinicopathological Correlations

We calculated the weighted sum of 31 m6A-associated lncRNAs obtained by multivariate Cox regression and named it risk score, a new prognostic characteristic (Therneau and Grambsch, 2000). The predictive validity of the risk model was verified by survival analysis, a risk plot, and receiver operating characteristic (ROC) curve. The prognostic capabilities of the predictive model for 1/3/5-year OS were evaluated by ROC curves (“timeROC” package) and the area under the curve (AUC) values (Blanche et al., 2013). The value of AUC is the size of the area under the ROC curve. Univariate and multivariate Cox regression analyses were performed to verify the independent prognostic role of risk score. The abovementioned analyses were performed by R software 4.0.2.

### Statistics

The statistical analyses were performed using the R programming language (R version 4.0.2) and GraphPad Prism 8. Kaplan–Meier curves and the log-rank (Mantel–Cox) test were used to compare the OS among three subgroups based on the expression of m6A-related lncRNAs. The Pearson correlation coefficient was used to reflect the degree of linear correlation between two random variables. The Wilcox test was used to compare the differences in gene expression and risk scores between subgroups. In all analyses, a two-tailed *P* < 0.05 was considered statistically significant.

## Results

### The Landscape of m6A RNA Methylation Regulators in COAD Patients

In this study, we obtained a TCGA data set involving 473 COAD tissues and 41 normal tissues and a GEO validation data set involving 652 colon tumor samples and 27 normal samples.

**FIGURE 1:**
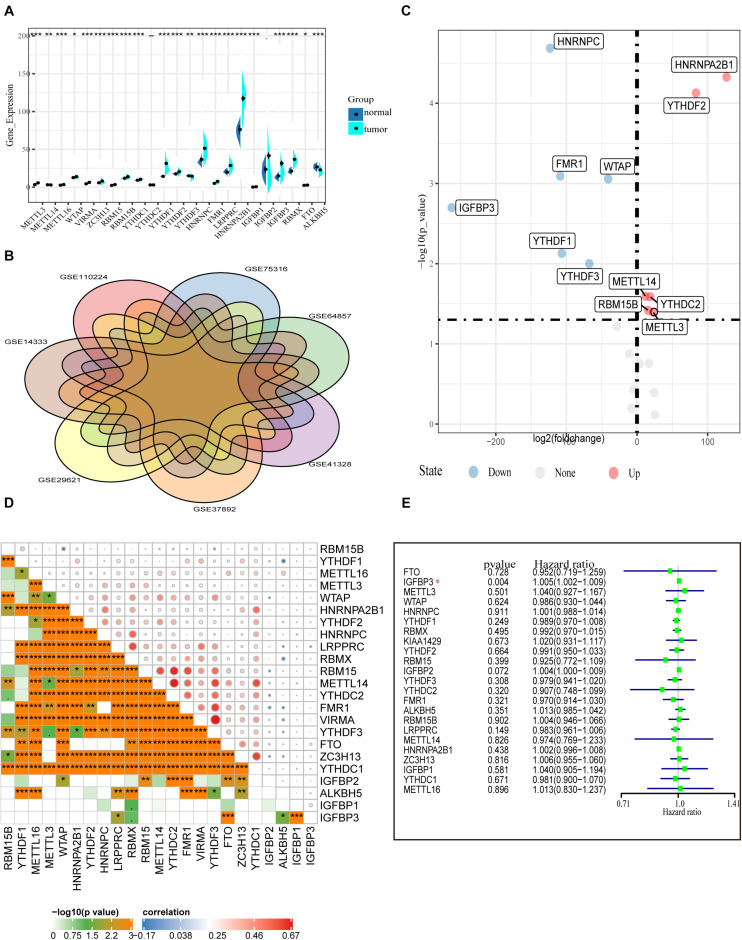
Transcriptome profiles of m6A RNA methylation regulators in COAD. **(A)** Differential expression of m6A RNA regulators between 473 tumor tissues and 41 normal tissues in the TCGA-COAD cohort. **(B)** The presence of batch effects in COAD data in the GEO data set. **(C)** Normalized GEO data for differential expression of m6A RNA genes between 652 tumor and 27 normal tissues. **(D)** The correlation of the m6A regulatory genes in the TCGA-COAD cohort. **(E)** Forest plot of the prognostic ability of the m6A RNA methylation regulators in the TCGA-COAD cohort.

First, the gene expressions of 23 m6A RNA methylation regulators were extracted by downloading RNA-seq data of COAD from the TCGA database (see text footnote 1), including eight m6A writers: METTL3, METTL14, METTL16, WTAP VIRMA, ZC3H13, RBM15, and RBM15B; 13 readers: YTHDC1/2, YTHDF1/2/3, HNRNPC, FMR1, LRPPRC, HNRNPA2B1, IGFBP1/2/3, and RBMX; and two erasers: ALKBH5 and FTO. Figure 1A shows the expression of various m6A RNA methylesterases. In comparison with normal colon tissues, the tumor tissues demonstrate generally higher expression of METTL3, METTL16, WTAP, VIRMA, ZC3H13, RBM15, RBM15B, YTHDC1, YTHDF1, YTHDF2, HNRNPC, FMR1, and LRPPRC. On the contrary, the expressions of HNRNPA2B1, IGFBP1 IGFBP3, RBMX, FTO, METTL14, and ALKBH5 were decreased in tumor tissues. However, there were no significant differences in the expression of YTHDF2 and IGFBP2 between tumor tissues and adjacent normal tissues. In particular, HNRNPA2B1 demonstrated the highest expression in tumors followed by HNRNPC and YTHDF1 (*P* < 0.01, Figure 1A).

To confirm the expression of m6A methylation factors in COAD, we downloaded seven colon cancer gene microarray sequencing data from the GEO database (see text footnote 2), including GSE110224, GSE14333, GSE29621, GSE37892, GSE41328, GSE64857, and GSE75316. First, we merged multiple data and performed the removal of batch effects using the combat function (“sva” package). Figure 1B shows the presence of batch effects among these data. With the normalized data of 652 colon cancer samples and 27 normal tissue samples, the mRNA levels of various m6A RNA methylation regulators were analyzed using the limma method. Similar to the results from the TCGA database, we found that METTL3, RBM15B, YTHDC2, YTHDF2, and HNRNPA2B1 were highly expressed in the tumor samples. Differently, HNRNPC, IGFBP3, FMR1, WTAP, and YTHDF1 expression were reduced in tumors according to the GEO database (Figure 1C). Besides this, Pearson correlation analysis was performed to assess the inter-regulatory effects between these m6A methylation regulators in TCGA-COAD. Figure 1D shows a close correlation between different m6A RNA methylation regulators. The demethylase ALKBH5 and the methylation-binding protein IGFBP family member IGFBP1/2/3 were negatively correlated with most of the other m6A regulators. The VIRMA gene and the YTHDF3 gene were most related. The YTHDF3 gene was most likely to be upregulated when the VIRMA gene was upregulated (Figure 1D). A univariate Cox regression analysis was used to identify the relationships between m6A regulators and the prognosis of COAD patients. Forest plots show that only IGFBP3 could be considered as a protective factor, and the other m6A RNA methylases were not relevant to the prognosis of COAD patients (Figure 1E). Moreover, we performed survival analysis for these 23 m6A RNA methylases using the TCGA database and the GSE29621 data set. However, we found no statistical significance of these genes in predicting survival (Supplementary Figure 1). These data indicate that, although m6A RNA regulators play crucial roles in the development and progression of COAD, it is still inadequate to predict the survival of colon cancer patients by m6A RNA methylation regulators alone.

### Interactions and Correlations Between m6A RNA Regulators and lncRNAs

**FIGURE 2:**
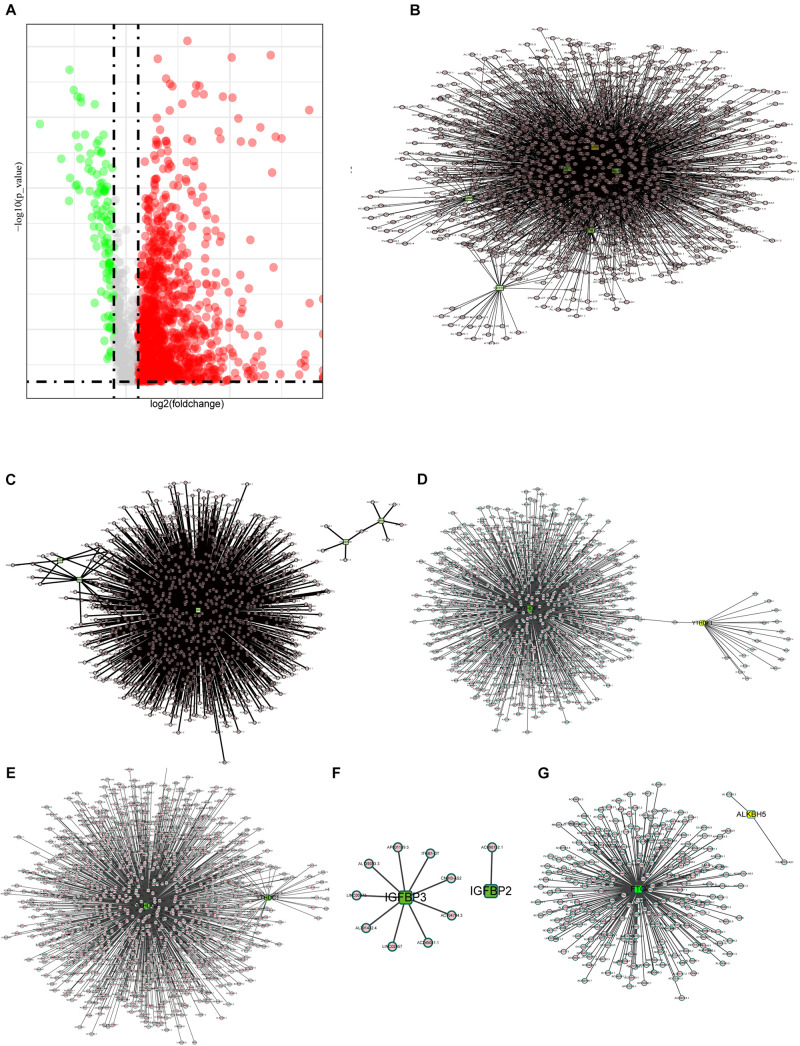
Interactions and correlations between m6A RNA regulators and lncRNAs. **(A)** Volcano plot showing differential expression of lncRNA regulators in the TCGA-COAD cohort. **(B)** Pearson correlation analysis was used to search the relationship between m6A regulators and lncRNAs. The regulatory network between m6A writers and lncRNAs demonstrated by Cytoscape. **(C–F)** Correlation between m6A regulator readers and lncRNAs expression, highlighting the YTHDF, YTHDC, and IGFBP families. **(G)** The regulatory network between m6A erasers and lncRNAs.

It is reported that m6A methylesterase regulates the RNA metabolism of lncRNAs, such as RNA splicing, RNA stability, etc., making lncRNAs dysregulated and, thus, contributing to important roles in tumor progression (Ma et al., 2019; Yang et al., 2020). Thus, the purpose of this study is to understand the role of m6A-related lncRNAs in COAD progression. First, we extracted the lncRNA expression matrix from the TCGA database and screened the differentially expressed lncRNAs. Figure 2A shows the differentially expressed lncRNAs with red dots representing genes highly expressed in tumor tissues, green dots representing genes highly expressed in normal tissues, and gray dots indicating no differential expression.

Here, we define lncRNAs associated with any of the m6A methylation regulators (| Pearson *R*| > 0.4 and *p* < 0.001) as m6A methylation-associated lncRNAs, and we identified 1,582 m6A-related lncRNAs. The results are demonstrated in Figures 2B–G, which shows the intricacy of these regulatory networks. To demonstrate the correlation of the three m6A regulators with lncRNAs, we visualized the network of regulatory lncRNAs separately. In Figure 2B, as m6A writers, RBM15, METTL14, METTL3, VIRMA, WTAP, and ZC3H13 are all strongly associated with lncRNA regulation. In Figure 2C, FMR1 plays a dominant regulatory role in lncRNAs, and there are few lncRNAs associated with RBMX and LRPPRC. In Figure 2D, the YTHDF family regulatory lncRNA network shows that YTHDF3 is found to play an important role in regulating lncRNAs, but no relevant lncRNAs are found for YTHDF2. The lncRNAs associated with YTHDC3 are absent, and YTHDC2 is correlated with many lncRNAs, implying that YTHDC2 has an important role in regulating lncRNAs. The IGFBP family seems to be less correlated with lncRNAs; only nine lncRNAs are correlated with IGFBP3, whereas only lncRNA AC090152.1 is correlated with IGFBP2 (Figure 2E). Among the m6A erasers, the ALKBH5-related lncRNAs were only two, much less than FTO, which might imply a potential role of FTO in regulating lncRNAs (Figure 2G).

### Prognosis Analysis of the m6A-Related lncRNAs

**FIGURE 3:**
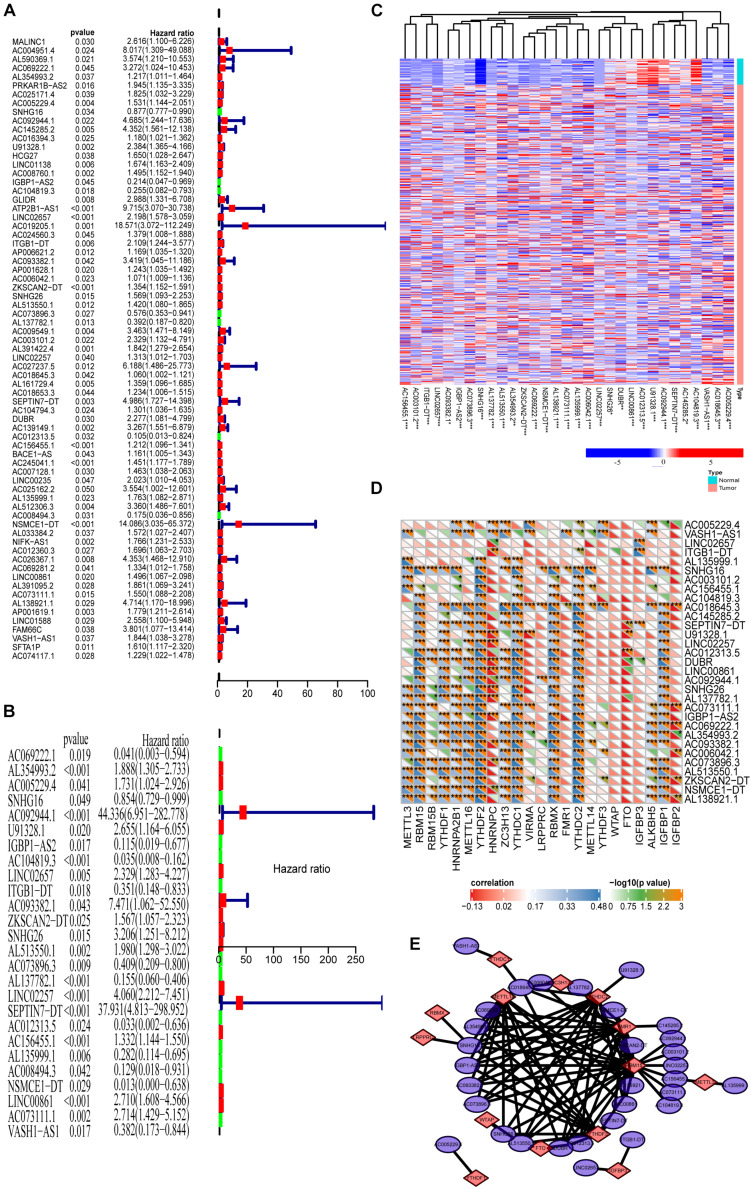
Prognosis analysis of the m6A-lncRNAs. **(A,B)** Univariate and multivariate analyses reveal the prognostic ability of the m6A-related lncRNAs. **(C)** Heat map showing the expression levels of 31 prognostic m6A-related lncRNAs in the TCGA-COAD cohort (Wilcoxon test). **(D)** Heat map of the correlations between m6A-related genes and the 31 prognostic m6A-related lncRNAs. **(E)** The regulatory network between m6A methylated genes and 31 lncRNAs with prognostic significance.

Because we reveal the close correlations between m6A RNA methylation regulators and lncRNAs in COAD, we next uncover the prognostic values of these m6A-related lncRNAs in COAD. Our previous results show that m6A RNA methylation regulators alone cannot adequately predict the prognosis and survival status of COAD patients. Considering that lncRNAs play essential roles in tumorigenesis and development, we integratedly analyzed the m6A-regulated lncRNA with prognostic value. First, univariate Cox regression shows that 66 of 1,582 lncRNAs are significantly correlated with prognosis (Figure 3A, *P* < 0.05). Multivariate Cox regression shows that there are 31 lncRNAs significantly correlated with the prognosis of COAD (Figure 3B, *P* < 0.05). Figure 3C demonstrates these prognosis-related m6A-lncRNAs expressions in COAD using a heat map. The correlations between these lncRNAs and m6A-related genes are shown in Figure 3D. As shown in Figure 3E, the correlation between 31 lncRNAs with prognostic significance and the corresponding m6A methylation genes is summarized by a network graph.

### Consensus Clustering of m6A-Related lncRNAs With Prognostic Value in COAD Patients

**FIGURE 4:**
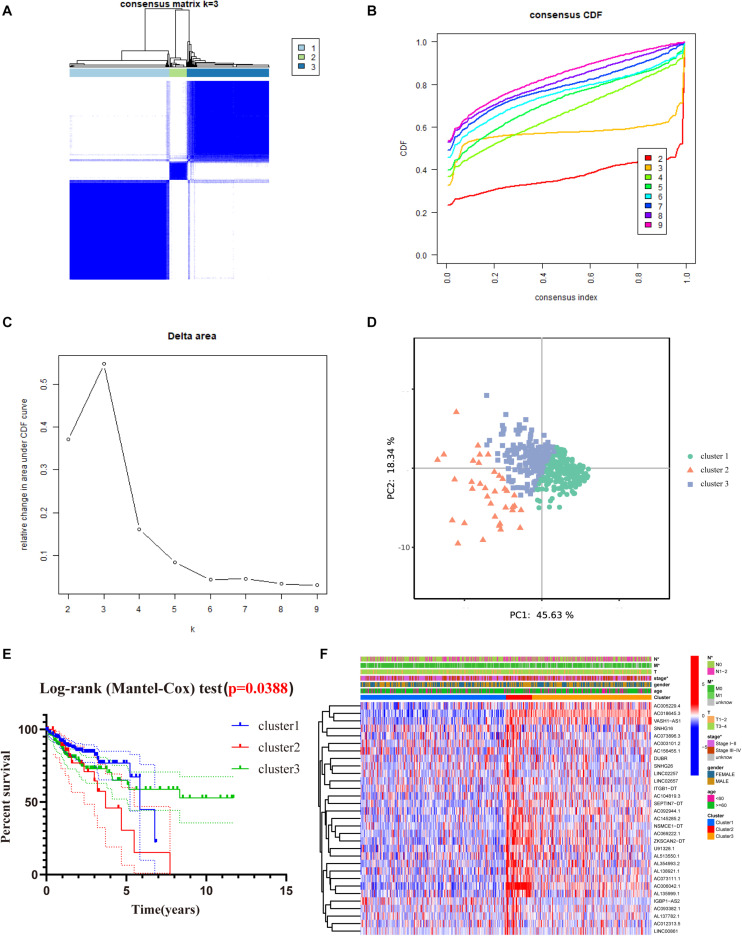
Consensus clustering of the tumor cohort of TCGA-COAD based on 31 prognostic m6a-related lncRNAs. **(A)** Consensus clustering matrix for *k* = 3. **(B)** The empirical CDF plot for *k* = 2 through 9. **(C)** Relative change in area under the CDF curve for *k* = 2 through 9. **(D)** PCA shows that, when *K* = 3, it is possible to well distinguish COAD into three clusters based on the isoforms determined by the expression of prognostic m6A-related lncRNAs. **(E)** Kaplan–Meier curves of OS for three clusters in COAD. **(F)** Heat map of the association between the m6a-related lncRNAs from three clusters and clinicopathological features in the TCGA data set (*P < 0.05).

To better understand the role of m6A-related lncRNAs in the development of COAD, consensus clustering was used to group COAD patients based on the expression of 31 prognosis-associated m6A-related lncRNAs. Figure 4A shows the matrix heat map for *k* = 3, called CM plots, which reveal the classification effect between the three clusters. The empirical cumulative distribution function (CDF) plot displays the common clusters for *k* = 2 through 9, intending to find the *k* for which the distribution reaches an approximate maximum, indicating maximum stability (Figure 4B). Figure 4C illustrates the delta area plot, in which the delta area score (*y*-axis) indicates the relative increase in cluster stability. Generally, the elbow method was used to take the value of *k* at the inflection point, which is the best classification number. Together, we classified COAD patients into three clusters. Furthermore, we performed PCA to compare the consistency of our samples with different clusters, showing distinctly different characteristics of the three clusters (Figure 4D).

To evaluate the clinicopathological characteristics between the three clusters, a survival analysis was performed. As shown in Figure 4E, cluster 2 had the worst survival status with a roughly 35% 5-year survival rate, and cluster 3 had nearly a 75% survival rate (Figure 4E, *P* = 0.038). To figure out whether there were associations between the different subgroups and clinical features, a heat map reveals that the *N*-classification, stage, and metastasis of the patients demonstrate significant differences between different clusters (Figure 4F).

### Potential Biological Functions of the Three Clusters

**FIGURE 5:**
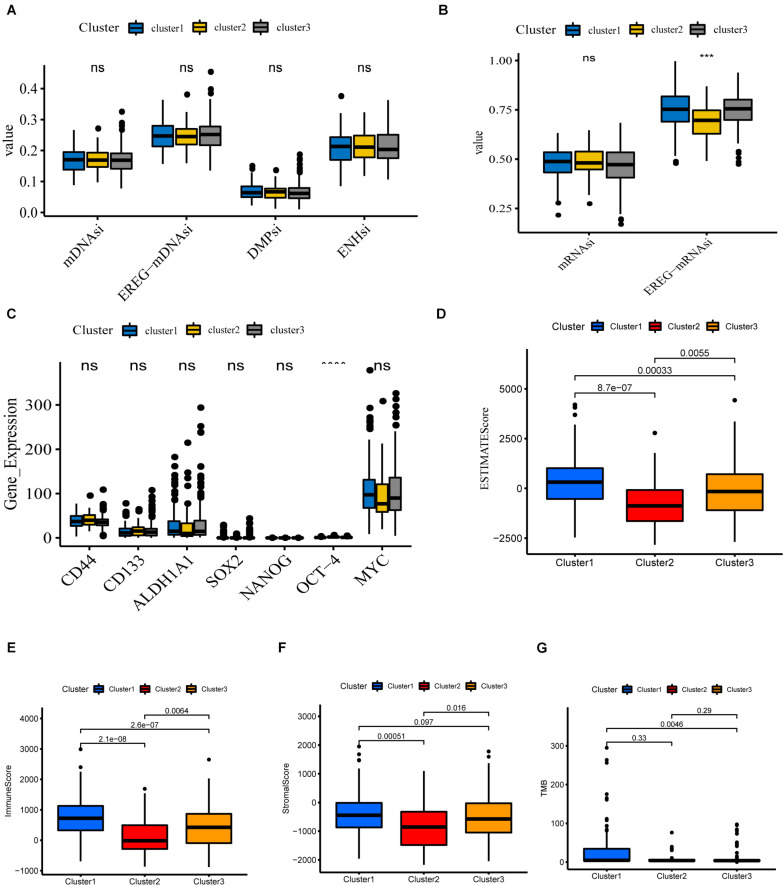
Potential biological functions of the three clusters. **(A,B)** Differential expression of stemness indices among three clusters, including mRNAsi (based on gene expression) and mDNAsi (DNA methylation-based stemness index) in the TCGA-COAD cohort. **(C)** Differential expression of relevant stem cell markers in COAD among three clusters. **(D–G)** Different expression of ESTIMATEscore, Immunescore, Stromalscore, TMB in three clusters (****P < 0.0001; ***P < 0.001).

Cancer stem cells, described as those with self-renewal capacity and capable of producing heterogeneous tumor cells in tumors, contribute to tumor proliferation, metastasis, drug resistance, and recurrence (Chang, 2016; Zhou et al., 2018). To quantitate the stemness of tumors, Malta et al. (2018) develop a predictive model using the OCLR algorithm to calculate stemness indices for TCGA pan-cancer samples. The workflow to produce the stemness indices is available at https://bioinformaticsfmrp.github.io/PanCanStem_Web/. There are two types of stemness indices. One is based on gene expression, including mRNAsi and EREG-mRNAsi (mRNAsi regulated by epigenetics). Another category is the DNA methylation-based stemness index mDNAsi, available as DMPsi, ENHsi, and EREG-mDNAsi (Malta et al., 2018). We first compared the stemness indices between these three subgroups. As shown in Figures 5A,B, only EREG-mRNAsi was differentially expressed, which was not yet sufficient to claim the difference of stemness degree between clusters. Munro et al. (2018) summarize the current stem cell markers used to identify and localize CSC subpopulations in colorectal cancer. Based on this, we investigated the expression levels of these genes between different subgroups. The results are shown in Figure 5C; for example, CD44, CD133, and ALDH1A1 do not differ between different clusters. Taken together, these results show that cluster analysis is not accurate for assessing the degree of tumor stemness.

We next investigate the differences in immune function. The ESTIMATE algorithm was used to generate StromalScore and ImmuneScore for all COAD samples. StromalScore, ImmuneScore, and ESTIMATEScore between the different clusters are presented using box plots (Figures 5D–F). We find that the three clusters show similar trends in StromalScore, ImmuneScore, and ESTIMATEScore. Cluster 1 demonstrates the highest scores, followed by cluster 3, and cluster 2 shows an immune deficiency. These results suggest that m6A-related lncRNAs are closely correlated to the tumor immune microenvironment. Besides this, the tumor mutational burden (TMB) is considered a promising indicator for predicting the response to immune checkpoint inhibitors, closely related to immunity. We analyzed the TMB values of COAD samples and found significant differences between clusters 1 and 3 (Figure 5G).

### Immune Landscape in COAD Patients

We further investigate the role of m6A-related lncRNAs in the immune function of COAD. For this purpose, we evaluated the correlation between m6A-related lncRNAs and immune genes as well as tumor-infiltrating immune cells, which would serve as powerful indicators to assess the tumor immune microenvironment.

**FIGURE 6:**
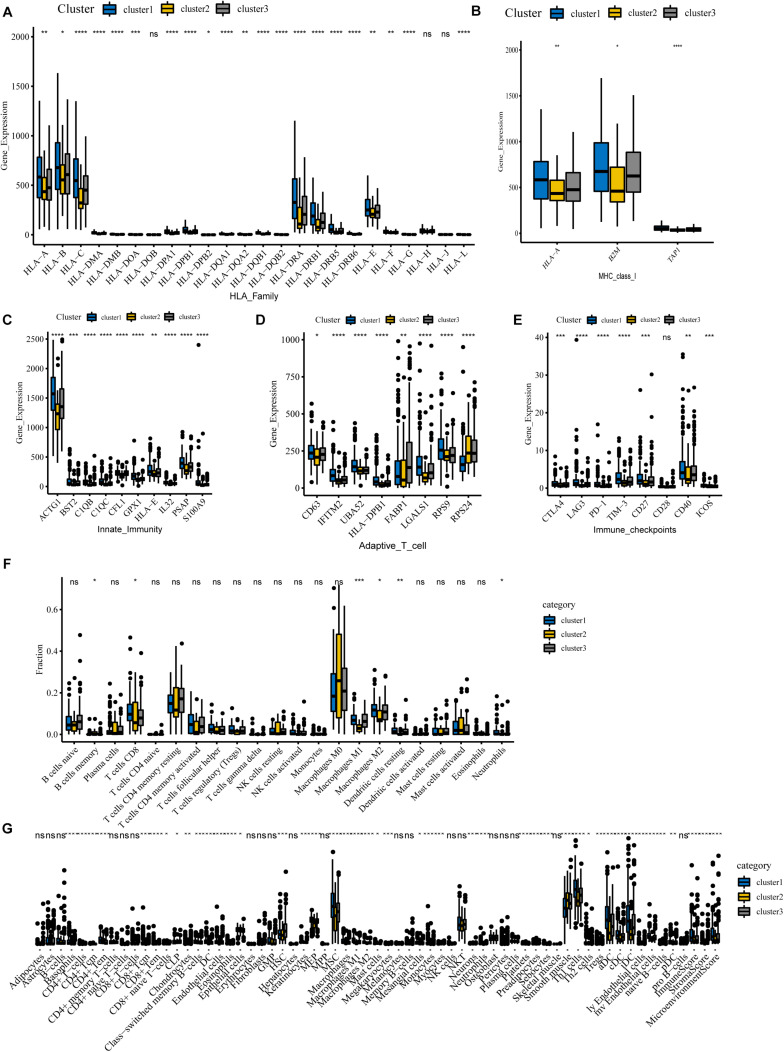
Immune landscape among three m6A-lncRNAs patterns. **(A,B)** According to the TCGA-COAD tumor cohort, the total transcriptome profile of immune genes was analyzed for differences among subgroups. Differential levels of HLA family genes **(A)** and MHC-I molecules **(B)** between the clusters. **(C–E)** Differential expression of innate immunity associated genes **(C)**, activation of T cells **(D)**, and relevant immune checkpoints **(E)**. **(F)** Differences in the infiltration levels of 22 immune cell types in three m6A patterns using the CIBERSORT algorithm. **(G)** Differences in the levels of infiltration of the 64 immune cells in three clusters using the Xcell website (****P < 0.0001; ***P < 0.001; **P < 0.01; *P < 0.05).

To begin with, we questioned whether there were any differences in immune genes between different clusters. We downloaded immunologic signature gene sets from the Msigdb website and extracted immune genes to examine their expression in three subgroups. Our results show that the genes of the HLA family are significantly differentially expressed in different clusters (Figure 6A). Human leukocyte antigen (HLA) is a major histocompatibility complex (MHC) expression product in humans, an antigen-presenting molecule, and regulates the immune response (Wang C. et al., 2020). In cluster 1, HLA-A, HLA-B, HLA-C, HLA-DRA, HLA-DRB1, HLA-E, and other genes were notably more highly expressed than in cluster 2, implying a robust immune response in cluster 1 patients and a deficiency of immune function in cluster 2. Figure 6B reveals that MHC-I molecules, such as HLA-A, B2M, and TAP1 are highly expressed in cluster 1, which further confirms our conclusion. Also, we detected the expression of immune-related genes and found that only genes involved in innate immunity and activated T cells differed between clusters. Our results show that levels of these genes are lowest in cluster 2, implying that cluster 2 is poorly immunized in intrinsic immunity and activated T cells (Figures 6C,D). Interestingly, many negative regulatory receptors, also known as immune checkpoints or co-inhibitory receptors, are expressed on T cells only after activation (Saibil and Ohashi, 2020). Several studies outline the immune checkpoint molecules involved in colorectal cancer development (Julie et al., 2015; Emambux et al., 2018); therefore, we compared the levels of relevant immune checkpoints between different subgroups. As shown in Figure 6E, the expression levels of immune checkpoints, such as PD-1, CTLA-4, and LAG-3, were the lowest in cluster 2, and cluster 1 demonstrates the highest expression levels, which implies that cluster 1 might have a better response to immunotherapies.

For tumor-infiltrating immune cells, we used two different algorithms to analyze them. First, we compared 22 different immune cell types in various clusters using the CIBERSORT algorithm. The results show that B cells, CD8 T cells, memory CD4 T cells, activated CD4 T cells, macrophages M0, macrophages M1, macrophages M2, and mast cells account for a large proportion of the immune cell infiltrates (Figure 6F). Cluster 1 with the best ImmuneScore showed an increased number of B cells, CD8 T cells, memory CD4 T cells, activated CD4 T cells, and M1 and M2 macrophages compared to cluster 2, which had the lowest ImmuneScore, indicating an activated immune system in cluster 1, whereas cluster 2, with more M0 macrophages, had a deficient immune status. Furthermore, another algorithm of Xcell was also used to visualize the immune microenvironment in COAD patients. The algorithm calculated 64 types of immune infiltrating cells. Similar to the CIBERSORT algorithm results in Figure 6G, tumor immune infiltrating cells exhibited significant differences between the different subgroups. Compared with the immune-deficient cluster 2, an increasing number of immune cells, including natural killer cells, activated TH1 cells, TH2 cells, and dendritic cells, were present in cluster 1. Notably, there were fewer stromal cells in cluster 1. Our results suggest that m6A-related lncRNAs could suppress or enhance the infiltration of immune cells, which could potentially affect the response to immunotherapy. In conclusion, our consensus clustering analysis well exhibited the immune status among different patients and could serve as a method to assess immunotherapy response in COAD patients.

### Construction of a Novel Prognostic Risk Signature for COAD

**FIGURE 7:**
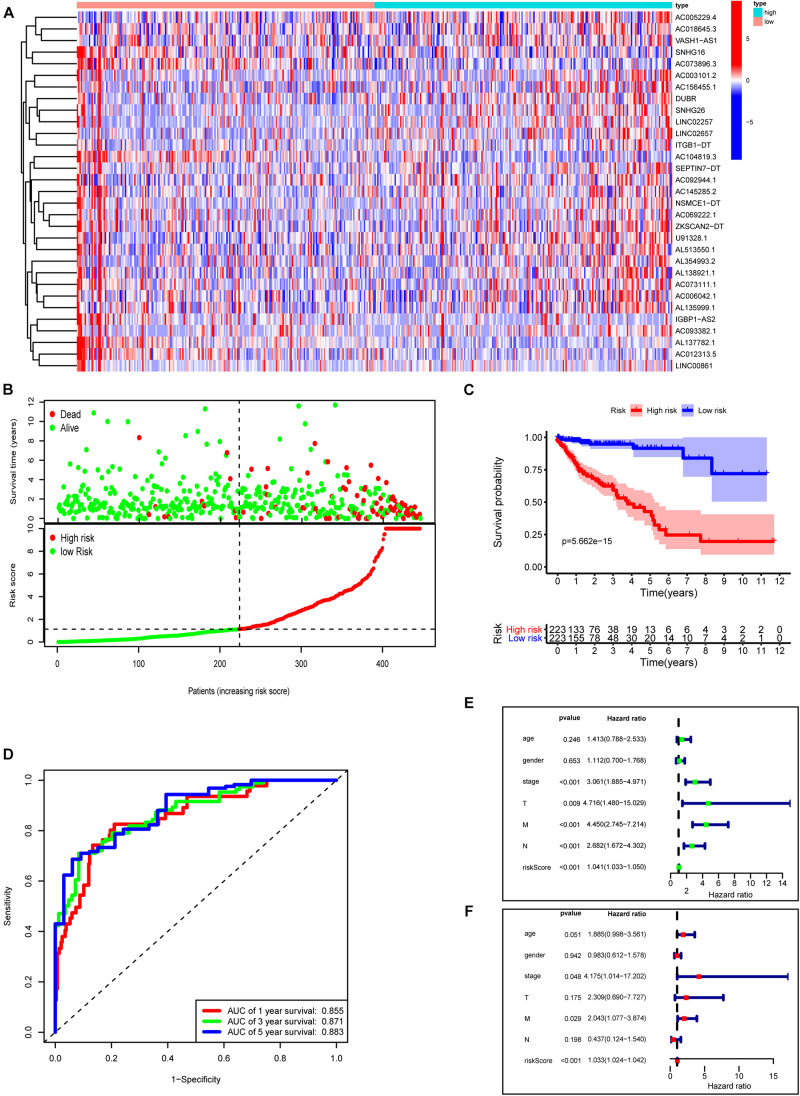
Construction of a novel prognostic risk signature for COAD. **(A)** Heat map of the associations between the expression levels of the 31 m6A-related lncRNAs and risk scores in the TCGA data set. **(B)** Risk plots showing the distribution of risk scores and life/death status. **(C)** Kaplan–Meier curves showing that the low-risk subgroup had better OS than the high-risk subgroup in the TCGA-COAD cohort. **(D)** ROC curves of risk scores for predicting the 1/3/5-year survival in the TCGA data set. **(E)** The association between clinicopathological factors (including the risk score) and overall survival by univariate Cox regression analysis. **(F)** The association between clinicopathological factors (including the risk score) and overall survival by multivariate Cox regression analysis.

Next, we used these 31 prognosis-related m6A-lncRNAs to construct the risk model with the coefficient of these lncRNAs to calculate the risk score as described in “Materials and Methods.” Patients were divided into low- and high-risk subgroups by the medium value of the risk score for further evaluation. Figure 7A shows the expressions of these 31 m6A-related lncRNAs in the high- and low-risk groups, respectively. Figure 7B plots the distribution of risk scores and survival status with each dot representing a sample and those in red indicating death. Survival analysis shows that COAD patients in the high-risk group have poorer clinical outcomes with a 5-year survival rate of only roughly 40% compared with approximately 90% in the low-risk group (Figure 7C, *P* < 0.0001). ROC curves show good sensitivity and specificity of the risk model in predicting survival status in TCGA-COAD (1-year AUC = 0.855, 3-year AUC = 0.871, 5-year AUC = 0.883; Figure 7D). We used univariate and multivariate Cox analyses to assess whether risk score was an independent prognostic factor for COAD patients. Univariate Cox analysis revealed that risk score was significantly associated with OS (hazard ratio: 1.041, 95% confidence interval: 1.033–1.050, *p* < 0.001, Figure 7E). Multivariate Cox analysis further showed that risk score was an independent predictor of OS (HR: 1.033, 95% confidence interval: 1.024–1.042, *p* < 0.001, Figure 7F). Risk scores were significantly associated with many clinical-pathological features (Supplementary Figure 2). Risk scores increased as the stage and TNM classification of the tumor advanced. Together, our risk model constructed with m6A-lncRNAs demonstrates good predictive performance in assessing the clinical prognosis and survival status.

## Discussion

With the advanced stage and poor OS, treating COAD is a substantial clinical challenge that requires new therapeutic targets (Xie et al., 2020). m6A modifications, accounting for the majority of RNA methylation, regulate tumorigenesis by modifying mRNAs and lncRNAs. m6A-based RNA modifications affect almost all biological functions of tumor cells (Lan et al., 2019; Yi et al., 2020). For example, m6A reader YTHDF2-modified lncRNA FENDER degradation significantly promoted endometrioid endometrial carcinoma cell proliferation (Shen et al., 2021). LNCAROD enhanced its mRNA stability through m6A methylation modification, and a complex with HSPA1A and YBX1 promoted the progression of head and neck squamous cell carcinoma (Ban et al., 2020). LNC942 directly recruited METTL14 protein through specific recognition sites, thus enhancing the expression of downstream target genes CXCR4 and CYP1B1 and promoting breast cancer cell proliferation (Sun et al., 2020). METTL3-modified lncRNA pseudogene Olfr29-ps1 can pass the Olfr29-ps1/miR-214-3p/MyD88 regulatory network to promote the immunosuppressive function and differentiation of mononuclear macrophages (Shang et al., 2019). However, until now, the potential role of m6A-regulated lncRNAs in COAD prognosis is unclear. In this paper, we focus on the expression, prognostic value, and immune significance of m6A-related lncRNAs in COAD, which could guide our future research directions.

First, we analyzed the expressions of 23 m6A genes in the TCGA-COAD cohort and found that most m6A genes had significant alterations compared with normal tissues, such as METTL3, METTL16, WTAP, and HNRNPA2B1. To further confirm the expression of these m6A methylation factors, we integrated seven colon cancer data sets from the GEO database. We found that METTL3, RBM15B, YTHDC2, YTHDF2, and HNRNPA2B1 were indeed highly expressed in the tumor samples. Meanwhile, there were strong interconnections between these different m6A regulators. VIRMA and YTHDF3 were the most correlated, and YTHDF3 was most likely to be upregulated when VIRMA was upregulated. However, univariate Cox regression analysis based on the TCGA and GSE29621 data sets shows that most m6A RNA methylases were not associated with the prognosis of COAD patients. These results suggest that the current m6A regulators remain inappropriate for predicting prognosis in COAD.

Several studies report that m6A-related lncRNAs are associated with tumor development. The establishment of prognostic models with m6A-related lncRNAs had a good performance in predicting tumor prognosis (Tu et al., 2020; Wang H. et al., 2020; Wang W. et al., 2020; Xu et al., 2021). For example, Zewei Tu develops a prognostic model consisting of nine m6A-related lncRNAs in patients with low-grade gliomas (Tu et al., 2020). m6A-related lncRNAs were potential biomarkers for predicting prognosis and immune response in patients with LUAD (Xu et al., 2021). In gastric cancer, the m6A-related lncRNA signature could serve as a novel prognostic factor (Wang H. et al., 2020). Therefore, exploring the role of m6A-related lncRNAs in the prognosis or diagnosis of COAD would contribute to the understanding of the molecular mechanisms of COAD. However, until now, the role of m6A-related lncRNAs in COAD has not been investigated, which deserves further research.

We filtered the differentially expressed lncRNAs from the TCGA-COAD cohort and analyzed their correlations with m6A regulators, yielding 1,582 m6A-related lncRNAs. Among the m6A readers, there were more lncRNAs related to FMR1 than other readers. Among m6A erasers, FTO was strongly correlated with multiple lncRNAs, which might imply a potential role of FTO in regulating lncRNAs. Next, univariate Cox and multivariate Cox analyses identified 31 m6A-related lncRNAs with prognostic values, which were used to establish a m6A-related lncRNAs prognostic signature. These lncRNAs did demonstrate moderate-to-strong correlations with m6A-related genes.

Moreover, the significance and potential underlying biological functions of these m6A-related lncRNAs were investigated. Patients were classified into three clusters according to consensus clustering analysis to facilitate the study of potential biological functions. It is generally accepted that tumor stemness and the immune microenvironment are two major contributors limiting the prognosis and treatment efficiencies of cancer patients (Saygin et al., 2019; Toor et al., 2019). Thus, we focus on the correlations of m6A-related lncRNAs with tumor stemness and tumor immunity. We found that many stem cell markers and stemness indices demonstrated no differences between subgroups, which suggests that these m6A-lncRNAs are inappropriate for assessing the degree of tumor stemness.

Studies concerning the immune microenvironment show the important roles of immunotherapies in COAD (Koi and Carethers, 2017; Zhang et al., 2018). In our study, the ESTIMATEScore, especially the ImmuneScore, demonstrated significant differences among these clusters, which reveals that these m6A-lncRNAs play important roles in the tumor immune microenvironment. Meanwhile, we observed a significant difference in TMB between clusters 1 and 3. Therefore, we further investigated the role of m6A-related lncRNAs in the immune function of COAD. For this purpose, we compared immune genes and tumor-infiltrating immune cells among different clusters, which serve as a powerful indicator to assess the tumor immune microenvironment. HLA is involved in antigen molecule delivery during cellular immunity and plays an essential role in the antitumor immune mechanism (Wang C. et al., 2020). We observed that the expression levels of the HLA family and MHC-I molecule genes were significantly higher in cluster 1 than in cluster 2. Meanwhile, genes involved in natural immunity and activation of T cells were highly expressed in cluster 1 along with increased expression of immune checkpoints, which confirmed the robust immune response in cluster 1, whereas cluster 2 patients lacked immune function. We used different algorithms to analyze tumor-infiltrating immune cells in COAD. Similarly, immune infiltration cells (B cells, CD8 T cells, memory CD4 T cells, activated CD4 T cells, and M1 and M2 macrophages) were significantly reduced, and M0 macrophages were increased in the low ImmuneScore group (cluster 2) compared with the high ImmuneScore group (cluster 1 or 3). These results suggest that a comprehensive assessment of m6A-related lncRNAs would help us to understand the characteristics of immune cell infiltration and might predict the response to immunotherapy.

We constructed a risk model consisted of 31 prognosis-related m6A-associated lncRNAs to calculate the risk scores of COAD patients. Based on the median risk score, COAD patients were divided into low- and high-risk subgroups with the high-risk group demonstrating poor survival status. Survival analysis, ROC curves, and univariate and multivariate Cox regression analyses show that risk score is a reliable independent prognostic indicator for COAD with significant relationships among many clinical-pathological features. The increasing risk score with advancing clinical grade not only suggests that the expression of m6A-lncRNAs may promote tumor progression, but also demonstrates the importance of risk score in predicting the prognosis of COAD. Notably, our prognostic risk model could obtain higher accuracy with AUC values > 0.8 vs. previous prognostic indicators (clinical stage). In conclusion, our m6A-related lncRNA model might serve as a new potential and promising biomarker that could provide more accurate clinical applications and valid treatment guidelines for COAD.

In conclusion, this study is the first comprehensive identification and systematic analysis of m6A-related lncRNAs in COAD. We identify m6A-related lncRNAs with prognostic value and construct a novel risk model with good predictive performance for prognosis and survival status. The risk score is highly correlated with the malignant clinicopathological features of COAD and can be regarded as a new potential and promising biomarker. Never before has clustering analysis of m6A-related lncRNAs been reported to exert essential roles in the immune and TME of COAD. Our results provide important evidence for further studies indicating the function of m6A-related lncRNAs in COAD, which could provide new insights into the guidance of effective immunotherapy in COAD.

## Data Availability Statement

The datasets presented in this study can be found in online repositories. The names of the repository/repositories and accession number(s) can be found in the article/Supplementary Material.

## Author Contributions

GL and LL designed the study. PZ performed the data analysis, graphing, and writing. LL was responsible for the critical reading of the manuscript. All authors read and approved the final manuscript.

## Conflict of Interest

The authors declare that the research was conducted in the absence of any commercial or financial relationships that could be construed as a potential conflict of interest.

## Footnotes

1 https://portal.gdc.cancer.gov/

2 https://www.ncbi.nlm.nih.gov/geo/

3 http://sangerbox.com/Tool

4 https://www.gencodegenes.org/human/

5 http://www.bioinformatics.com.cn

6 https://bioinformaticsfmrp.github.io/PanCanStem_Web/

7 http://www.gsea-msigdb.org/gsea/msigdb/collections.jsp#C7

8 https://cibersortx.stanford.edu/

9 https://xcell.ucsf.edu/#

## Supplementary Material

Supplementary Figure 1 Survival analysis of m6A RNA methylation regulators in colon cancer patients.

Supplementary Figure 2 Risk scores were significantly associated with many clinical-pathological features.

## Abbreviation

COAD: colon adenocarcinoma
lncRNAs: long non-coding RNAs
m6A: N6-methyladenosine
OS: overall survival
TCGA: The Cancer Genome Atlas
GEO: Gene Expression Omnibus Database
TME: tumor microenvironment
HR: hazard ratio
ROC: receiver operating characteristic
AUC: area under the curve
EFS: event-free survival
TMB: tumor burden
PCA: principal components analysis
CSC: cancer stem cells
HLA: human leukocyte antigen
MHC: major histocompatibility complex
CDF: cumulative distribution function.
