# Supplementary figures and images for: N6-Methylandenosine-Related lncRNA Signature Is a Novel Biomarkers of Prognosis and Immune Response in Colon Adenocarcinoma Patients
Source: Front Cell Dev Biol. 2021 Jul 15;9:703629. doi: 10.3389/fcell.2021.703629 (PMC8321625; doi:10.3389/fcell.2021.703629)

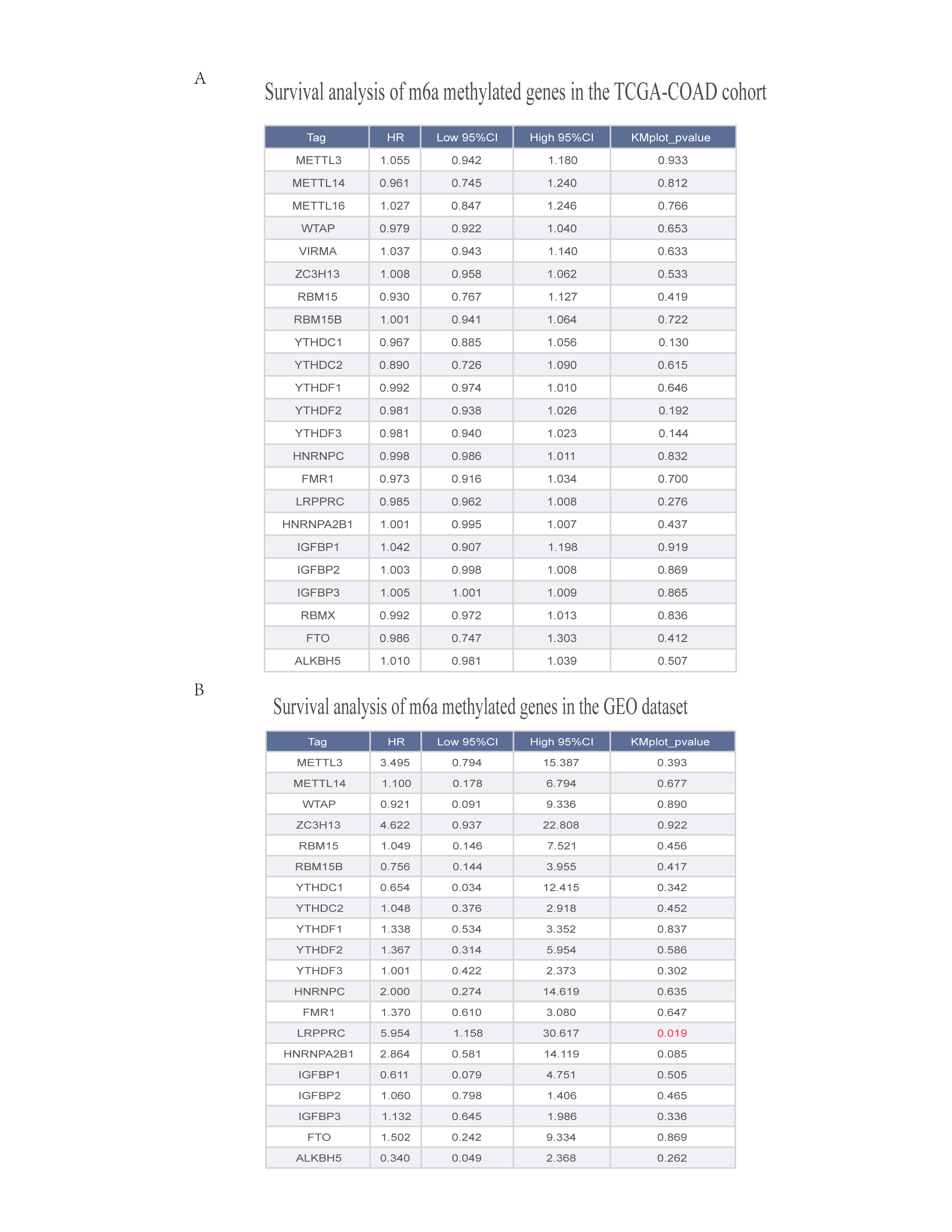

Supplement: Supplementary Figure 1 — Survival analysis of m6A RNA methylation regulators in colon cancer patients. [file Image_1.TIF]

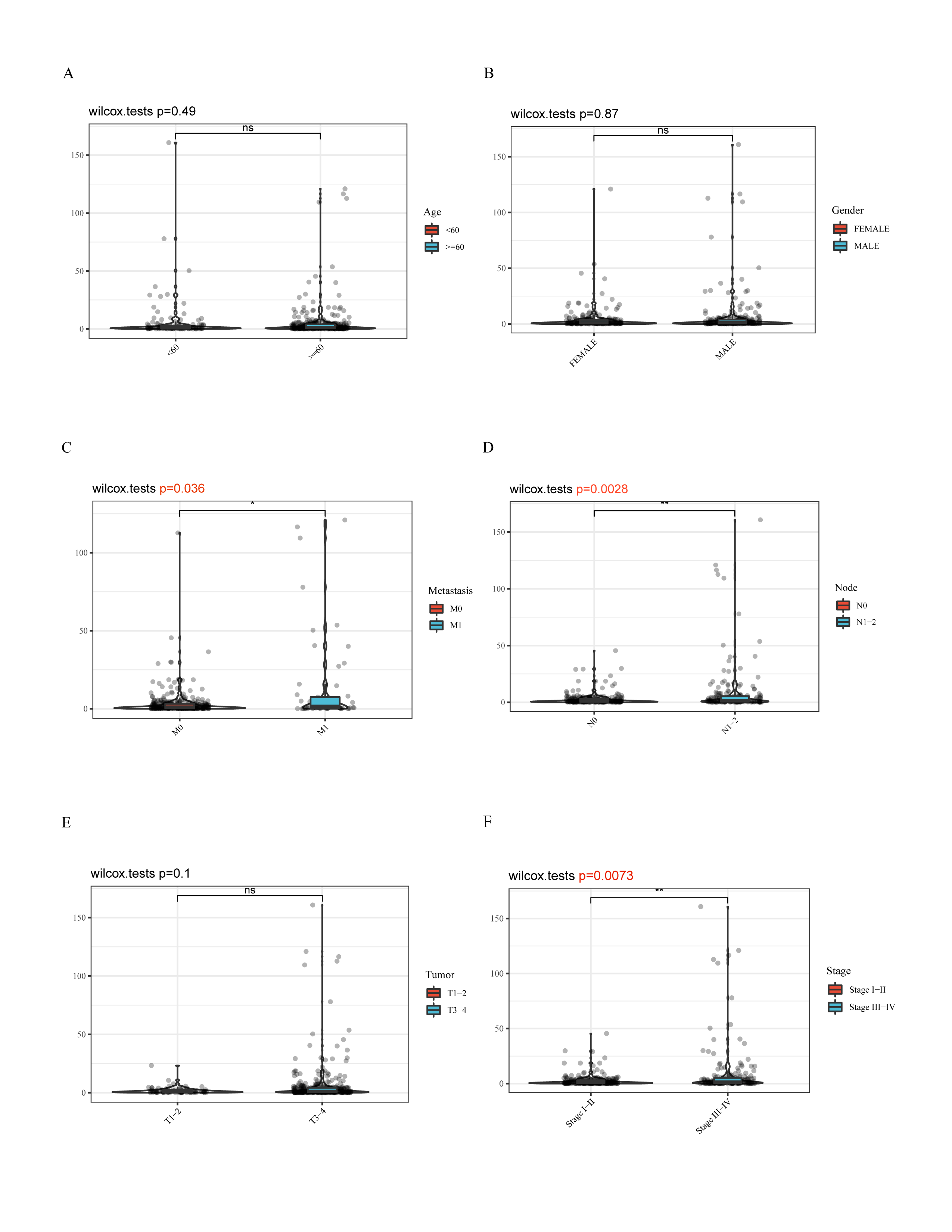

Supplement: Supplementary Figure 2 — Risk scores were significantly associated with many clinical-pathological features. [file Image_2.TIF]
